# Supplementary material for: Teaching deep networks to see shape: Lessons from a simplified visual world
Source: PLoS Comput Biol. 2024 Nov 11;20(11):e1012019. doi: 10.1371/journal.pcbi.1012019 (PMC11581402; doi:10.1371/journal.pcbi.1012019)
Supplement: S1 Appendix — To demonstrate the robustness of our findings, we show results from additional experiments with a more complex dataset and with pretrained networks, as well as additional training runs with a vision transformer with a wider range of hyperparameters. (PDF) [file pcbi.1012019.s001.pdf]

Appendix: Additional experiments
Teaching deep networks to see shape: Lessons from a simplified visual world.

Christian Jarvers[1*], Heiko Neumann[1]

1 Institute for Neural Information Processing, Ulm University, Ulm, Germany

* christian.jarvers@uni-ulm.de

1 Additional experiments with a larger stimulus set

We designed our datasets with the goal to isolate the feature dimensions of interest – shape, color, and texture – as much as possible, resulting in very simple stimuli. While this simplicity facilitates our analysis, it comes with the risk of reduced validity: it is possible that there are qualitative differences in how networks learn on our simple datasets, compared to more complex, naturalistic image datasets. For example, networks may fail to learn to use shape as a feature because the training set was too small, had too few classes, or included too little variation between different shapes.

To mitigate these concerns, we repeated experiment 1 (testing for bias on a dataset with two features) and experiment 2 (testing for learnability on a dataset with a single feature) on a larger dataset with a larger set of shape classes. Specifically, we generated a dataset with 10 classes, where each class was defined by the two features shape and color. We used 5 basic shape classes (rectangles, ellipses, crosses, triangles, and parallelograms), each of which could be either horizontal (width larger than height) or vertical (height larger than width), resulting in a total of 10 shape classes. For the color feature, we took 20 equally spaced base hues in the HSV color space and randomly assigned two of these hues per class. For each image, we sampled a HSV value by adding some random noise to the hue value and sampling random S and V components. We generated 1000 images per class, varying position, size, and aspect ratio randomly across images. Fig A shows example images of the different classes.

We used the same training protocol as in experiments 1 and 2 (see Methods). Then we tested the performance on a test set with the same shape- and color-classes as the training set, but with independently sampled positions, color values, etc. In order to test whether networks relied on color or shape features, we used two additional test sets. In the random shape test, the color classes were the same as in the training set, but each image had a completely random shape. In the random color test set, the shape classes were the same as during training, but the color of each image was chosen randomly.

Fig B shows the test results. Each network was able to learn the task and achieved over 90% accuracy. The two transformer networks *ViT-B-16* and *Swin-T* performed equally well on the random shape test set, but just below chance level (10%) on the random color test set, indicating that their classification performance is based on color only. In contrast, the convolutional networks *VGG-19* and *ResNet-50* performed above chance level on all test sets. However, the performance on the random shape test set was lower than on the standard test set and the performance on the random color test set was even lower. This indicates that the convolutional networks learned a mixture of shape and color features, but relied more strongly on color than on shape.

This differs from our results in experiment 1, in which none of the networks showed evidence of shape selectivity. On the multi-shape dataset, the networks succeeded in learning some shape features. This may be because the new multi-shape dataset has a

Figure A: **Example images from multi-shape dataset.**

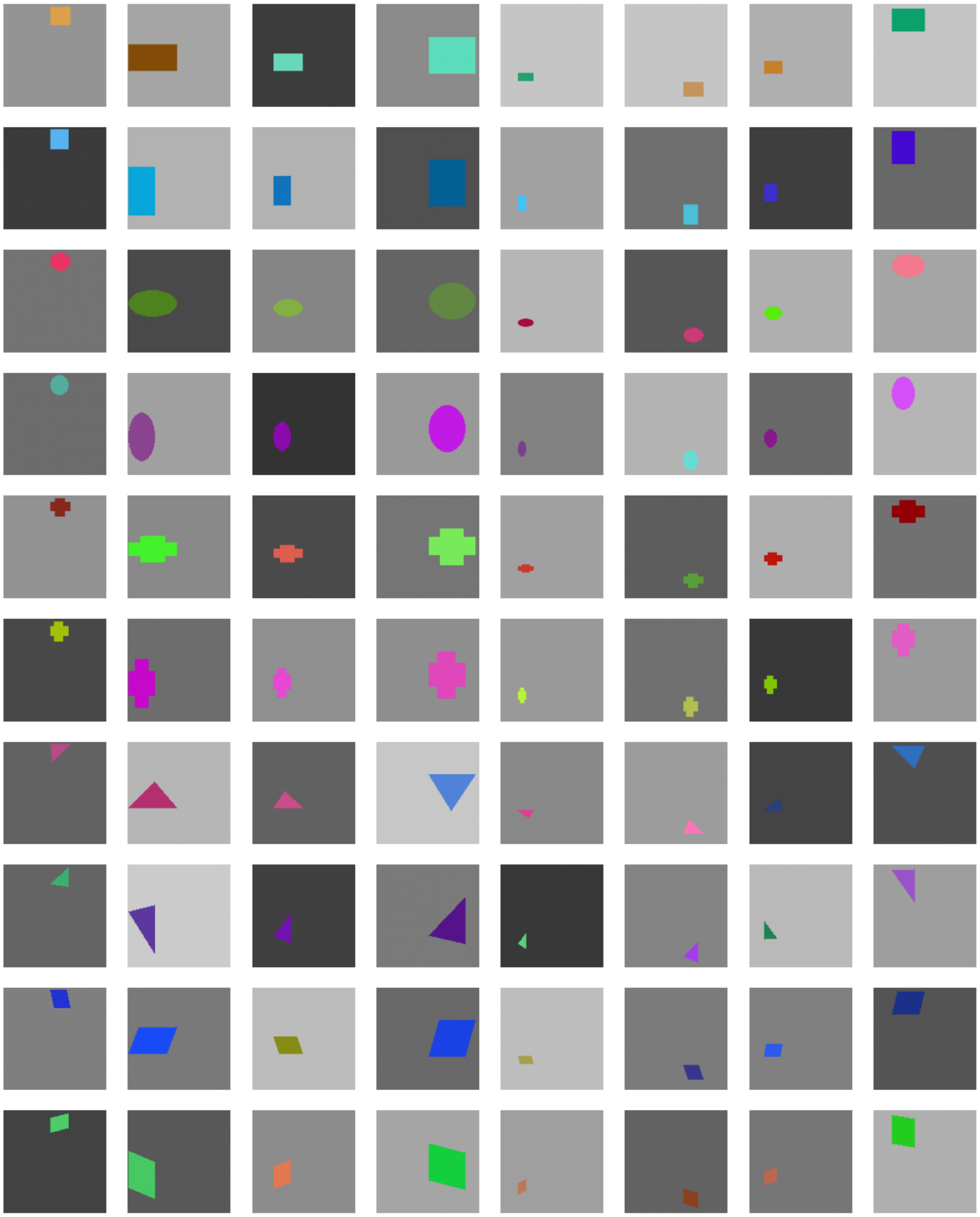

Each row shows example shapes from one class. For example, the first row contains images from the horizontal rectangle class, whereas the fourth row contains images from the vertical ellipses class.

richer set of shape features. For example, the rectangle and L-or-T datasets only contained right angles. In contrast, classes in the multi-shape dataset can be distinguished by the angles they contain: triangles only contain acute angles, rectangles only right angles, parallelograms a mix of acute and obtuse angles, and crosses both right and reflex angles, whereas ellipses contain no angles.

This observation opens up the question why some shape features are easier to learn and others are more difficult. Previous work showed that deep networks can learn

Figure B: **Performance on multi-shape dataset.**

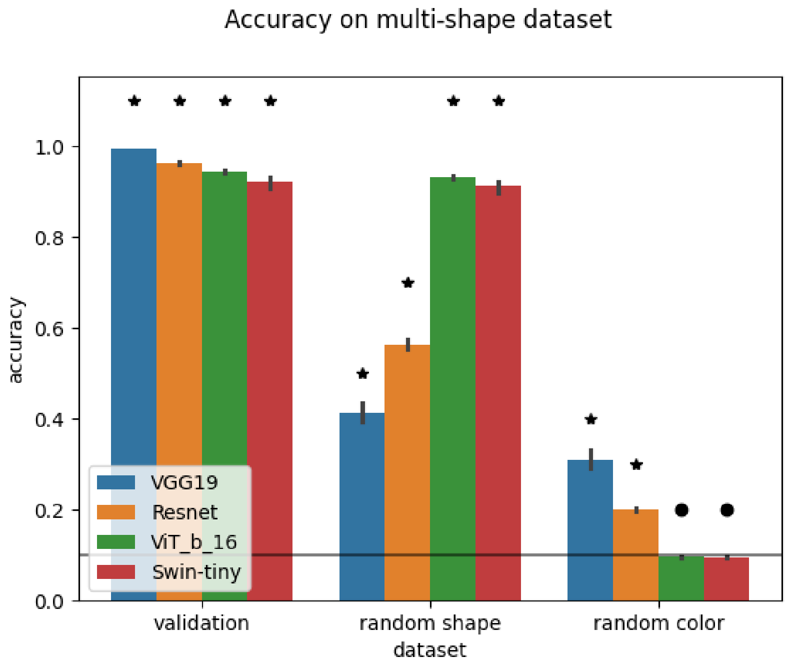

Accuracy of standard networks after training on multi-shape dataset. In the training and test sets, both features (shape and color) were equally predictive of the image class. In the random shape test set, only color predicted the image class. In the random color test set, only shape predicted image class. Error bars indicate 95% confidence intervals estimated from 10 training runs with different random initial weights. Stars indicate that accuracy is significantly above chance level (horizontal line) according to a sign test. Circles indicate that accuracy is significantly below chance level.

local shape features, but are less sensitive to global shape features [1, 2] such as the overall arrangement of image parts, and spatial relations between parts of an object [3]. Our results could be interpreted in a similar fashion: our datasets in experiments 1 to 4 rely on global shape features (horizontal vs. vertical rectangles) and relational features (L and T are distinguished by whether the second bar attaches to the end or middle of the first one), which are both difficult to learn. In contrast, the multi-shape dataset contains both local shape features (acute vs right vs obtuse angles) and more global features (horizontal vs. vertical orientation).

Therefore, we also performed a learnability experiment (similar to experiment 2) with the multi-shape dataset. Specifically, we tested whether the standard networks were able to learn the classification task in four modified conditions with restricted feature sets:

1. **Random color**: the same ten shape classes as in the multi-shape dataset are used, but the color of each image is drawn completely at random, so color does not predict image class.

2. **Random shape**: the same ten color classes as in the multi-shape dataset are used, but the shape in each image is drawn randomly, so shape does not predict image class.

3. **Ignoring orientation**: there are only five shape classes (rectangles, ellipses,

crosses, triangles, and parallelograms). The orientation of the shape does not matter. The color of each image is drawn randomly and does not predict image class.

4. **Ignoring shape type**: there are only two shape classes - horizontal and vertical. The type of shape does not matter, only the orientation. The color of each image is drawn randomly and does not predict image class.

With these modifications to the dataset, we predict the following outcomes. If a network is generally unable to learn shape features, it should be able to learn the random shape task, but none of the others. A network that can only learn local shape features should be able to learn on the random color dataset and on the dataset that ignores orientation, but it should fail to learn the classification ignoring shape type.

Figure C: **Performance on modified variants of multi-shape dataset.**

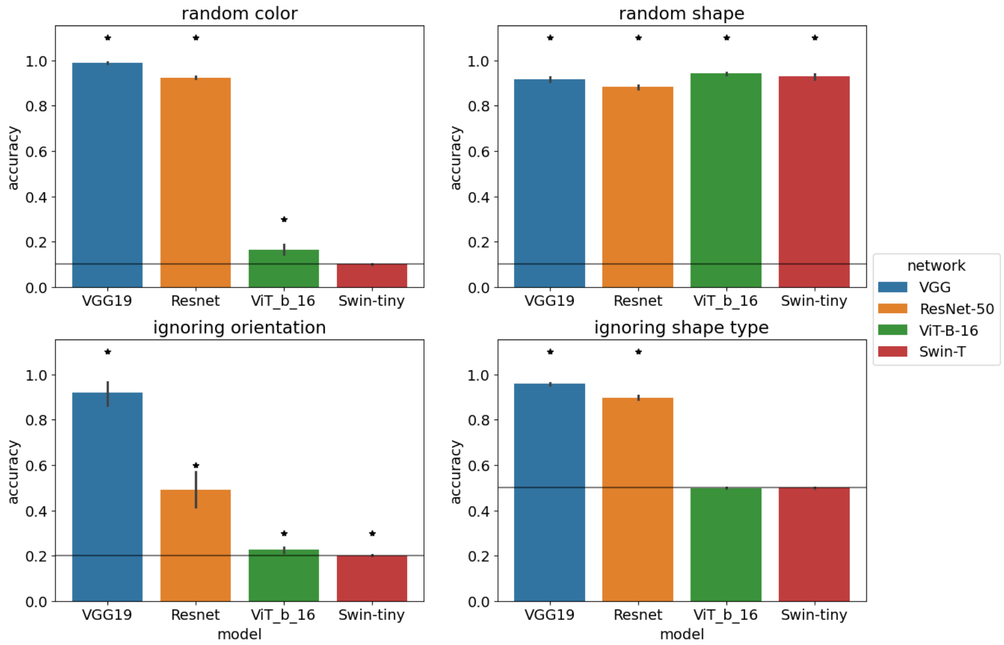

Accuracy of standard networks after training on variants of the multi-shape dataset with restricted features and testing on an independently generated version of the same datasets. Error bars indicate 95% confidence intervals estimated from 10 training runs with different random initial weights. Stars indicate that accuracy is significantly above chance level (horizontal line) according to a sign test.

We used the same training protocol as in experiment 2. The results are shown in Fig C. The convolutional networks *VGG-19* and *ResNet-50* are above chance level on all four datasets, indicating that they can use all feature types. This is consistent with our results in experiment 2. In contrast, the transformer-based networks *ViT-B-16* and *Swin-T*achieve a high accuracy only on the random shape dataset. On the random color dataset, *Swin-T* performs at chance level (10%) and *ViT-B-16* only slightly higher (16.5%). When ignoring orientation, both perform slightly above chance level (*ViT-B-16* at 22.6%, *Swin-T* at 20.1%, chance level is 20%). As in experiments 1 and 2, we used sign tests to check whether the number of training runs in which a network achieved an accuracy above chance level was higher than expected under the null hypothesis that the network answered randomly (see Methods). While these tests

indicate that the differences from chance are significant, it is unclear whether this really reflects that the networks learned the underlying classes, or whether they exploited accidental imbalances in our dataset. This would be in line with our results in experiment 2, in which transformers failed to learn shape-based classification.

Notably, these results do not show a difference between local and global shape features. The fact that *VGG-19* and *ResNet-50* are able to learn some shape features on the multi-shape dataset (Fig B) is not simply due to the presence of local shape features.

In summary, the results on the multi-shape dataset are consistent with our interpretation of experiments 1 to 4. This shows that our results generalize to slightly more complex datasets. Nevertheless, the multi-shape dataset is still far from natural images. Closing this gap is an interesting direction for future work.

2 Additional experiments with pretrained networks

One of the main difficulties in interpreting the results of experiments 1 to 4 comes from the simplicity of our artificial training images. Since we trained all networks from scratch, the networks likely learned to use very different features than they would on natural images with more variety and complexity. For example, there is a risk that networks failed to learn shape features on our datasets simply because there were not enough different features available. Testing this directly is not feasible, since we cannot control the available features in natural images to the same degree as in our artificial image datasets. However, we can approach this question by using networks that were pretrained on natural images.

Thus, we repeated experiment 1 (bias) and experiment 2 (learnability) with the standard networks (*VGG-19*, *ResNet-50*, *ViT-B-16*, and *Swin-T*) with weights pretrained on ImageNet. We used the pretrained weights provided by the `torchvision` library (version 0.16). In each case, we replaced the last fully-connected layer of the network with a new, fully-connected layer with 2 output classes and randomly initialized weights. We froze all pretrained weights and only trained the new, final layer. We used the same setup and training protocol as in experiments 1 and 2.

The results for training on the two-feature datasets are shown in Fig D. Similar to experiment 1, the networks largely show the pattern of results expected after learning color or texture, but not shape: they had high accuracy on the original training data and the color-/texture-only test sets, and performed significantly below chance level on the conflict test set. However, in contrast to experiment 1, *VGG-19* and *ResNet-50* performed above chance level on all shape-only test sets. This indicates that they learned some shape features. On the striped rectangles dataset, *Swin-T* also performed significantly above chance level on the shape-only test set. However, its average accuracy was 50.5%, only 0.5% above chance level, so it is possible that this result is an artifact and does not reflect real shape selectivity.

The results for training on the single-feature datasets are shown in Fig E. In contrast to experiment 2, all networks were able to learn both tasks. This is especially notable in the case of the transformer architectures *ViT-B-16* and *Swin-T*, which failed to learn shape-based classification in all other experiments. This shows that there exists a set of weights (reflecting features included in the data used for pretraining) with which these architectures can solve the task, but training from scratch fails to find it.

As we argued in the main text, this may be because the self-attention operation used in transformers can be better understood as similarity-based grouping [4], which fails on our shape-only datasets due to their extreme simplicity. Since the images only show simple grey figures in front of a grey background, all image patches are projected

Figure D: **Shape bias of pre-trained networks.**

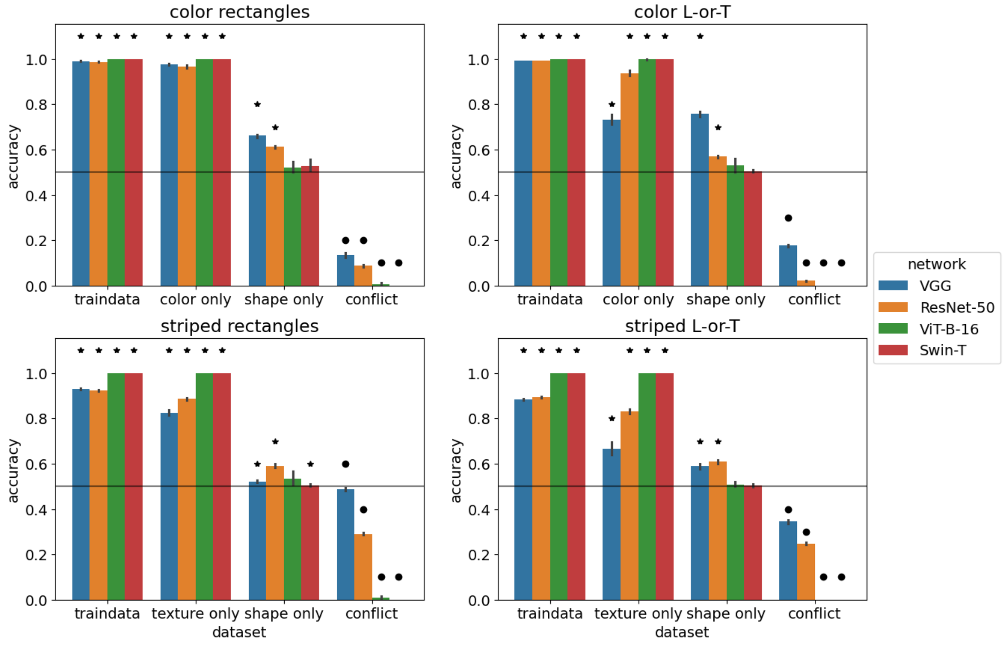

Accuracy of standard networks after pre-training on ImageNet and fine-tuning the last layer on our artifical images. Images in the training set had both features (shape and color / texture). Error bars indicate 95% confidence intervals estimated from 10 training runs with different random initial weights. Stars indicate that accuracy is significantly above chance level (horizontal line) according to a sign test. Circles indicate that accuracy is significantly below chance level.

Figure E: **Performance of pre-trained networks trained on shape-only images.**

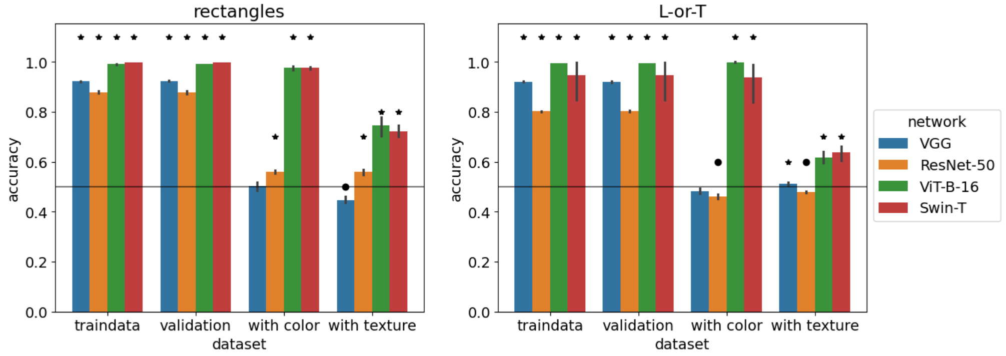

Accuracy of standard networks after pre-training on ImageNet and fine-tuning the final layer on our shape-only training sets. Error bars indicate 95% confidence intervals estimated from 10 training runs with different random initial weights. Stars indicate that accuracy is significantly above chance level (horizontal line) according to a sign test. Circles indicate that accuracy is significantly below chance level

onto relatively similar embeddings. This leads to mutually high attention scores, which result in even more similar representations after each attention block. Effectively, since all image areas are similar, they are grouped together homogeneously,

leaving no differences to distinguish the classes by.

Pre-training on ImageNet alleviates this issue because ImageNet contains natural images with many different features. This leads to the formation of more selective projection weights in the embedding and attention layers, which amplify the small differences between image patches in our dataset (the contrasts at the shape border). Therefore, with the pre-trained weights enough information is transported to the final layer to enable it to learn the classification task.

3 Additional training runs with vision transformers

In experiment 2, all transformer architectures were unable to learn the shape-only classification tasks. Given the success of the transformer architecture on a wide range of tasks, this failure is surprising. In addition, after pre-training on ImageNet, *ViT-B-16* and *Swin-T* were able to learn both shape-only classification tasks (see Fig E). Thus, there is a set of weights that solves the task, but our training procedure fails to find it.

This failure to learn could simply be due to the fact that we used suboptimal hyperparameter settings for training, e.g., a wrong learning rate or too few epochs. While the same hyperparameters worked well on the full datasets, it is theoretically possible that learning shape-only classification requires a different hyperparameter set. To rule out this explanation, we re-ran the training of *ViT-B-16* on the shape-only rectangle dataset with a wider range of learning rates and for a larger number of epochs.

Figure F: **Performance of vision transformers with different hyperparameter settings.**

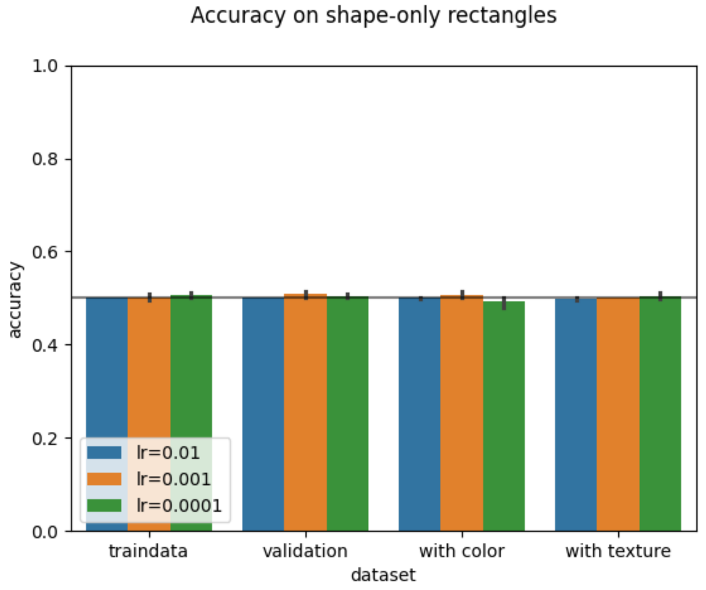

Accuracy of *ViT-B-16* after training on the shape-only rectangles dataset for 100 epochs with different learning rates. Error bars indicate 95% confidence intervals estimated from 10 training runs with different random initial weights. Accuracy did not significantly differ from chance (horizontal line) in any of the tests.

The results are shown in Fig F. The results are the same as in experiment 2: for all

hyperparameter settings, *ViT-B-16* fails to learn the task. While it is possible that there is some other combination of hyperparameters that results in learning, the fact that transformers consistently fail to learn this task for a wide range of hyperparameter settings and on different datasets (rectangles, L-or-T, and the multi-shape dataset discussed above) is sufficient evidence to conclude that this task is problematic for the transformer architecture. As we discuss in the main text, this may be due to the fact that transformers essentially work by grouping image elements by similarity [4] and that this grouping fails on our datasets due to the very simple nature of our images.

In addition to this observation about consequences of deprivation in training data, our findings might provide some guidance towards an enriched variation of feature dimensions. Training datasets like ImageNet have been previously criticised regarding their ecological validity. As a consequence [5] curated the *ecoset* data that more closely reflects the statistical relevance of perceptual and cognitive categories. The findings here point in a similar direction, but with respect to the structural level of input features. Specifically, it may be worthwhile to curate datasets in which feature dimensions related to shape are represented more richly. This could include curvature variations in magnitude and sign as well as additional and more densely sampled texture dimensions [6].

References

[1] Baker N, Lu H, Erlikhman G, Kellman PJ. Deep Convolutional Networks Do Not Classify Based on Global Object Shape. PLOS Computational Biology. 2018;14(12): e1006613. doi: 10.1371/journal.pcbi.1006613.

[2] Baker N, Elder JH. Deep Learning Models Fail to Capture the Configural Nature of Human Shape Perception. iScience. 2022;25(9). doi: 10.1016/j.isci.2022.104913.

[3] Malhotra, G, Dujmović, M, Hummel, J, & Bowers, JS Human shape representations are not an emergent property of learning to classify objects. Journal of Experimental Psychology: General. 2023;152(12): 3380–3402. doi: 10.1037/xge0001440

[4] Mehrani P, Tsotsos JK. Self-Attention in Vision Transformers Performs Perceptual Grouping, Not Attention. Frontiers in Computer Science. 2023;5. doi: 10.48550/arXiv.2303.01542.

[5] Mehrer J, Spoerer CJ, Jones EC, Kriegeskorte N, Kietzmann TC. An ecologically motivated image dataset for deep learning yields better models of human vision. PNAS. 2021;118(8): e2011417118.

[6] Rao AR, Lohse GL. Towards a texture naming system: identifying relevant dimensions of texture. Vision Research. 1996; 36(11): 1649-1669.
