# Supplementary material for: Teaching deep networks to see shape: Lessons from a simplified visual world
Source: PLoS Comput Biol. 2024 Nov 11;20(11):e1012019. doi: 10.1371/journal.pcbi.1012019 (PMC11581402; doi:10.1371/journal.pcbi.1012019)
Supplement: Attachment — Submitted filename: ResponseToReviewers.pdf [file pcbi.1012019.s002.pdf]

Response to Reviewers

First of all, we thank all three reviewers for their careful reading of the manuscript and for their kind and insightful comments together with their valuable suggestions. We believe that the additional experiments that were suggested considerably strengthen our results and that the suggested changes to the manuscript improve its readability.

Please find below point-by-point responses to the individual comments ordered according to each reviewer (our responses in blue, line numbers refer to the updated version with changes marked).

Reviewer 1

Comment: First, I think the two shape comparisons that the authors tasked the network with making have interesting differences from each other. The rectangle task involves sensitivity to a more global shape feature (aspect ratio), but the shapes are different from each other only because of their orientation relative to the image frame. The L's and T's differ in terms of their local features (for example, number of corners). A human observer spontaneously asked whether a horizontal and vertical rectangle have the same shape might say yes, but they would almost certainly not say yes for the L and T. I don't think the authors need to change the stimuli they used in any way, but it would be interesting to see the authors discuss how stimulus differences affected performance in their various experiments.

We agree that the distinction between local and global shape features is important, especially since previous work indicates that deep networks have difficulties processing global, but not local shape. However, we think that our stimuli are too simple and the results between the different shape datasets are too similar to allow any clear interpretation in this direction. We have added this point to the discussion in an additional paragraph which reads (lines 464ff.)

> To reduce the risk that our results only reflect the idiosyncrasies of our artificial stimuli, we designed datasets with two very different types of shape features. Distinguishing horizontal and vertical rectangles requires a global shape feature (aspect ratio), whereas distinguishing Ls and Ts is similar to the relational deformations explored in [36], but can be solved with local shape features (detecting L- and T-junctions). We also repeated our experiments with a slightly more complex dataset with more different shapes (rectangles, ellipses, triangles, crosses, and parallelograms) – see Supplement S1. The results were similar to experiments 1 and 2, demonstrating that the phenomena we describe here are not limited to our specific choice of datasets. The multi-shape dataset included both local shape features (such as different angles) and global features (aspect ratio) and we tested whether there were differences in which features the networks could learn (see Supplement S1, Fig S3). There were no clear differences between the different types of shape features, neither between the rectangle and L-or-T datasets, nor on the multi-shape dataset. In contrast, previous work showed that deep networks typically succeed at learning local shape features, but struggle with global shape [16, 19, 21]. This difference may be due to the simplicity of our datasets. While the distinction between horizontal and vertical shapes is global in the sense that it concerns the overall shape, it does not require detecting the correct spatial relation of multiple object parts.

Comment: A more minor suggestion would be for the authors to add a paragraph to their Experiment 1 Results section explaining spatial competition architectures a little more. This may be my ignorance, but I was not very familiar with them and was curious which of their properties may be conferring performance advantages in Exp. 1 and beyond. The authors do address this in the Discussion and give more detail in the Methods section, but knowing a little more about spatial competition architectures early on would be helpful for understanding the authors' findings.

We have followed this suggestion and added a brief explanation of the spatial competition architecture which now reads (lines 204ff.)

> The only network that showed evidence of shape selectivity was *spcConvNet*. This network differs from the other convolutional networks by incorporating spatial competition: nearby locations in each feature map compete for activation (see Methods). This suppresses activations in homogeneous areas, enhances activations at unique feature locations, and makes the network representations sparser.

We hope that this helps to contextualize and understand the results.

Comment: For Figure 7 and 9, I was a little confused about what the "labels" line was plotting. Could the authors clarify?

The "labels" line shows the cosine similarity between the NTK and a matrix that contains a 1 if two images belong to the same class and a 0 if they belong to different classes. We originally plotted this line for reference, but since it does not play a role in our analysis we decided to remove it, as suggested by Reviewer 2.

Comment: Another small concern I had was in the way the authors discussed the two possibly explanations for color/texture bias in lines 201-206. It seems that there is ample evidence that even ImageNet-trained deep networks learn some shape features and can classify by them. Otherwise, how else would DNNs perform better than chance on silhouette images? In my view, this does not preclude the possibility that there are kinds of shape features important to human perception that are DNNs to which DNNs are insensitive, such as more global and/or configural shape features. It would be helpful if the authors clarified their view on this.

We agree that there is clear evidence that deep networks can use some kinds of shape features. The second explanation (defect in architecture) is only meant to imply that there are some types of shape features that networks are unable to learn. We have rewritten the paragraph to clarify this point. It now reads (lines 224ff.).

> If networks are able to use the shape features in our datasets, their bias towards color and texture in experiment 1 is evidence for shortcut learning, supporting hypothesis 1. On the other hand, if networks are unable to learn to use the shape features in our datasets, this indicates a defect in network architecture, supporting hypothesis 2.[1]

with the footnote

> [1]Notably, an architectural defect does not have to mean that networks cannot learn to use any shape features, only that there are some shape features that networks are unable to learn. There is compelling evidence that deep networks can use some shape features, e.g., their ability to classify silhouettes. Nevertheless, there may be some shape features that they are generally unable to learn.

Comment: In a similar vein, I was a bit puzzled by the authors' finding that ViT was completely unable to learn to classify by shape. As the authors discuss in lines 405-406, this is inconsistent with previous findings that ViT significantly outperforms both VGG and ResNet in silhouette classification. Unless I misunderstood, I don't think the explanation they give in lines 406-419. What similarity-based grouping principles would allow for accurate silhouette recognition of animals in Baker & Elder (2022) but not for L's vs. T's?

We also agree that the inability of ViTs to learn in the shape-only condition is surprising. However, we do think that the findings of Mehrani & Tsotsos (2023), i.e., that transformers are best understood in terms of grouping by similarity, can serve as an explanation. In our previous draft, we only discussed this briefly and did not really account for the previous findings that ViTs have a higher shape bias than convolutional networks (Tuli et al., 2021) and that ViTs can classify silhouettes (Baker & Elder, 2022).

We have now expanded this discussion and hopefully made our explanation clearer. We also have taken the results of our additional replication experiments into account and discussed alternative interpretations for the failure of the transformers in our experiments. This part now reads (lines 507ff.)

> Surprisingly, all three transformer-based architectures that we tested completely failed to learn on the shape-based datasets when trained from scratch. In contrast, both *ViT-B-16* and *Swin-T* were able to learn the shape-only classification tasks after pre-training them on ImageNet and fine-tuning the last layer (see Supplement S1, Fig S5).

These results admit two very different interpretations. On one hand, it is possible that some aspect of our stimulus design or training setup interferes with how transformers learn. To test this, we repeated our experiments with a more complex dataset (see Supplement S1, Fig S3) and with a wider range of hyperparameters (see Supplement S1, Fig S6). Again, transformers were unable to learn classification tasks when only shape information was available. This shows that the underlying problem is at least somewhat robust. Nevertheless, it is possible that the transformers fail to learn the shape-only classification tasks not because of a deficiency in their shape processing, but because our images are too artificial and too simple. Alternatively, increasing the size of our dataset and models may make transformers more shape selective [37]. In this view, we cannot conclude anything about whether transformers can or cannot process shape in natural images.

We hope that this makes the discussion clearer and more balanced.

Additional comments:

Minor points:

- Line 12: "…how they have to be connected, to high-level…" should be "…how they have to be connected, and high-level…"
- Line 66: "…-why networks prefer rely on texture" should be "why networks rely on texture"
- Line 105: "…to lean shape-based classification" should be "to learn shape-based classification"
- Line 324: "…suggest that color and shape belong to the former kind of feature…" should be "…suggest that color and texture belong to the former kind of feature…"

We have corrected all passages listed above as suggested.

Reviewer 2

Comment: One of my key concerns is the clarity of the manuscript, especially Experiments 3 and 4. I believe most of the readers of this manuscript will (like me) not be familiar with Neural Tangent Kernels. While the authors have done a good job, especially in the Methods, to educate the reader, there are several aspects that are still quite obscure. Here are a few examples of statements / phrases that are difficult to make sense of without a deeper understanding of the NTK. I have included the difficulty in parsing these phrases within parentheses:

- l.259: "the evolution of the network function f(x;θ) follows the kernel gradient of the NTK" (but the reader may not know what a kernel gradient is)

  We agree with the reviewer's concern that the algorithm and function of NTK should be better explained to help readers better follow the logic of our investigations. As a consequence, we have substantially rewritten the paragraph containing this (and the following two) sentences. We have tried to eliminate jargon and clarify the explanations as far as possible. We hope this makes this section and the paragraphs therein easier to read and helps with understanding the subsequent explanations of our analysis.

- l.260: "This is especially useful in the limit of infinitely wide neural networks" (But the authors are not considering infinitely wide neural networks. So, it's unclear to me why the authors mention this.)

  Please see above.

- l.260: "at each point during the training the direction of the kernel gradient reflects what the network is learning [25], whereas the structure of the kernel reflects the features that the network has learned" (Why is there a dichotomy between the direction and structure of the gradient?)

  Please see above.

- l.287: "Why is the NTK more similar to the color- or texture-trained network?" (This is a shortening, right? My understanding of what you mean is: Why is the NTK of the network trained on the full dataset more similar to the NTK of the color- or texture-trained network, than the shape-trained network?)

  Yes, this is correct. We agree that this shortening is hard to understand and have written out the full sentence in the text. The more detailed explanation now reads (l.286ff revision)

  > Specifically, it can be thought of as a matrix with one coefficient for each pair of images x_i and x_j that describes how similar the gradients for these two images are. In this sense, the kernel describes a feature space in which some images are close to each other, while others are distant from each other. At the same time, the NTK describes a direction in the space of possible functions that the network can instantiate (with different parameters). During training with gradient descent, the network parameters θ are adjusted such that the network function f(x; θ) changes and moves through this function space. Crucially, the direction of this movement is exactly the direction described by the neural tangent kernel [25].

Comment: There are many other examples in the manuscript. I would recommend a substantial rewrite of Experiments 3 & 4 to clarify the language and only use jargon that is strictly necessary.

We have tried to simplify the language in the explanations for experiments 3 & 4 in several places. In addition, following the suggestion of Reviewer 3 we have added a simplified example at the end of the results for experiment 3. We hope that these steps make it easier to follow the results.

Comment: Related to point (1) above is clarifying some methods that the authors have used for the NTK analysis. This analysis is presented in a way that seems quite convoluted and I think many readers will find this impenetrable as there are a number of steps underlying this analysis. I would recommend that the authors present the overall structure of their argument somewhere early on in their manuscript, so that the reader knows where this is going. Here's my understanding of what this analysis involves: The analysis consists of four steps. First, the authors train three NTKs as reference NTKs. These are color-only NTK, texture-only NTK and shape-only NTK. Second, they compute a similarity (across all images in the datasets) between each of these NTKs and the NTK that learns on color+texture+shape. Frequently, they find a greater similarity with the color-only and texture-only NTKs. In Step 3, they cluster the images based on each NTK, varying the clusters from 2-50. In Step 4, they work out how well these clusters are "aligned" to the classes. The key result is that images clustered based on the color and texture NTKs align with class categories much better than those based on shape NTK. What does this show? According to the authors this shows that DNNs learn differently based on images that present the same shape but in different locations. In contrast, when the same color is presented in different locations, DNNs learn (adjust their weights) in a much more similar manner. In a mini-batch setting, this means that different images with same color lead to similar weight adjustment, while different images with same shape lead to different weight adjustments, which may cancel each other out. If my understanding is correct, this is a lovely and novel insight.

This summary of our analysis and interpretation is correct. We agree that summarizing the steps of our analysis early in the manuscript may help the reader follow the explanations. Therefore, we have expanded our explanation of the NTK analysis at the end of the introduction. This part now reads (lines 100ff.)

> We compare the neural tangent kernel of a network during training to reference kernels of networks with known feature selectivity and show that the direction of gradient updates is more aligned with color and texture features than with shape. To understand how this alignment arises, we analyze the structure of the NTK. Specifically, we identify groups of images which are similar to each other according to the similarity function expressed by the neural tangent kernel. This means that the network outputs for these images are related: changing how the network responds one image will also affect how it responds to others. Finally, we examine whether images in these groups tend to belong to the same class. We find that this is the case for NTKs of color- and texture- sensitive networks, but not shape-sensitive networks. We hypothesize that such groups dominate the dynamics of gradient descent learning, explaining why networks are biased to color and texture.

In combination with the adjustments for the previous point, this hopefully makes the NTK analysis much more accessible.

Comment: The other major shortcoming of the manuscript is the limited manner in which the authors have tested their hypotheses. There are several extensions to their study that may be useful and provide greater insight:

(i) One of the most surprising finding of the manuscript is that many standard network simply cannot learn some basic shapes. Many researchers will be unwilling to concede that the standard networks simply cannot learn shape. They may suspect, for example, this is due to the way the networks were tested - e.g. the size of datasets, the number of categories (2 categories might not be enough), hyper-parameters used (30 epochs may not be enough, the learning rule may not be appropriate, etc.) Therefore, it will be good to establish how robust these results are. Do they hold for other (larger) datasets? Do they hold for settings when dataset contains more than 2 categories? Do they hold for other settings of hyper-parameters (learning rate, number of epochs, regularization methods, learning rules)? To satisfy this sceptical reader, it will be good to carry out these tests.

We agree that these results are surprising and that additional experiments can help demonstrate the robustness of our findings. We have therefore carried out such replication experiments as suggested. This leads to several additional figures and accompanying explanations, which we felt would make the manuscript too long and interrupt the flow of our argument. We therefore decided to report these experiments in an appendix. Specifically, we have generated a new dataset with 10 shape classes and repeated our experiments. As in the original experiments, we found that transformers were unable to learn the shape-only version of this task.

We also carried out additional training runs with a vision transformer with different learning rate settings and more epochs. While there is still a possibility that the vision transformers would learn with some other hyperparameter setting, we think that the fact that they fail to learn (1) with various hyperparameter settings, (2) on several datasets, and (3) while they learn as soon as other features than shape are available, should be enough to convince a sceptical reader that these results are not just an artefact.

(ii) My own suspicion is that the networks are not learning these shapes as the shapes only differ in global properties (the relative width & height of sides, or the relationship between parts). This is a hard task for the network as they are far more likely to pick up local features (as shown by the results of Baker et al., Geirhos et al. and some of my own work - e.g. Malhotra et al.). If, however, there were local (shape) features that could distinguish the two categories, the standard networks should be able to learn them. For example, shape in one category had curved vertices, while the shapes in another category had sharp (angular) vertices (square vs circle vs triangle). If this is the case, then it would be incorrect to say that the networks cannot learn shape per se, but that they cannot learn global shape features but are capable of learning local shape features. It will be good to test this.

When we designed our datasets, we also thought that deep networks would most likely struggle with learning global shape features and successfully learn local ones. This was one of the reasons we used two types of shapes – we expected that the rectangle task would require a more global shape property (height vs width) and the L-or-T task would only require local shape features (L-junctions vs T-junctions). However, since our results did not show differences in the learnability of these two shape types (except for the ResNet), we did not stress this difference in our discussion.

We tried to test this again in our new experiments by designing the multi-shape dataset such that some classes are distinguished by local features (for example, rectangles and triangles differ in the acuteness of their angles), whereas others again differ by global features (there are horizontal and vertical versions of each shape). Again, we did not find any differences between global and local shape features. This may be because both our local and global shape features are very simple – too simple for differences in their learnability to become apparent.

Since previous work has shown the importance of the local-global distinction, we have included a brief discussion of this point in the manuscript (lines 464ff.) and in the supplement with the additional experiments.

(iii) The networks used by the authors have been trained from scratch. While this is useful, many studies have shown that pre-training the networks can lead the networks to acquire some biases. The authors can test this by downloading and using networks that have been pre-trained on various datasets, e.g. ImageNet and EcoSet.

We have repeated our experiments with networks that were pre-trained on ImageNet and included the results in the supplement. Networks showed slightly more evidence of learning shape features, but were still biased towards color overall. The largest difference was that all standard networks (including ResNet and the transformers) were able to learn the shape-only classification tasks. This is an interesting difference and we have included it in our discussion. However, we think that it does not invalidate our main argument.

(iv) The images in the dataset do not vary in scale. Are the network able to learn shape when scale is varied? This may seem counter-intuitive as one is increasing the size of the dataset and giving the network a harder problem to solve. But by making the problem harder, one may prevent the network getting stuck in local optima.

There is some variation in object scale in our datasets. For example, rectangles in the large version of the rectangle datasets vary in size from 20x10 to 60x49 pixels. In the new multi-shape dataset, the smallest objects are 11x7 pixels large, the largest are 56x54 pixels, so the object scale varies by a factor of about 5. We hope that this provides enough variety for network training.

Comment: Further experiment (optional): It is interesting to note that in Fig 7 the NTK similarity evolves for a short period and then reaches an equilibrium (at least in three out of four panels). This is likely because the network "discovers" color as a predictive feature and weight updates become smaller once color can predict nearly 100% of image classes. It is possible that shape is harder to discover than color and had the network not been able to discover color, or if color was less predictive than shape, then the network may have kept on learning shape features. The authors can test this by designing a slightly different experiment where color is less predictive than shape. For example, if color predicted output category for 50% of images but shape predicted output category for 100% of images, then it is possible that the network kept learning shape features and eventually the similarity with shape NTK increases.

This is an interesting idea. We agree that reducing the predictivity of the color/texture features would likely lead to the networks eventually learning shape, as was shown in Malhotra, Dujmovic and Bowers, 2022. It would be especially interesting to see whether the transition from a bias towards color/texture to shape is relatively gradual or more categorical. However, we have decided against including these experiments in the paper for two reasons: (1) the paper and appendix are already getting rather long and introducing and analysing these experiments would take considerable additional space, and (2) the outcome of this experiment would not substantially affect the interpretation of our other results.

Comment: Can the authors discuss how their results (showing that ViT cannot learn shape) square up with recent results that some vision transformers show the highest level of shape bias (Dehghani,..., Houlsby et al, 2023, ICML)?

We have included this point in our discussion. The main part that addresses this point reads (lines 508ff.)

> Surprisingly, all three transformer-based architectures that we tested completely failed to learn on the shape-based datasets when trained from scratch. In contrast, both ViT-B-16 and Swin-T were able to learn the shape-only classification tasks after pre-training them on ImageNet and fine-tuning the last layer (see Supplement S1, Fig S5).
>
> These results admit two very different interpretations. On one hand, it is possible that some aspect of our stimulus design or training setup interferes with how transformers learn. To test this, we repeated our

> experiments with a more complex dataset (see Supplement S1, Fig S3) and with a wider range of hyperparameters (see Supplement S1, Fig S6). Again, transformers were unable to learn classification tasks when only shape information was available. This shows that the underlying problem is at least somewhat robust. Nevertheless, it is possible that the transformers fail to learn the shape-only classification tasks not because of a deficiency in their shape processing, but because our images are too artificial and too simple. Alternatively, increasing the size of our dataset and models may make transformers more shape selective [37]. In this view, we cannot conclude anything about whether transformers can or cannot process shape in natural images.
>
> On the other hand, our results may indicate that the transformer architecture has a deficiency that prevents it from learning shape – at least the shape features present in our dataset – supporting hypothesis 2. After all, despite their simplicity our shape-only datasets did contain a feature (shape) and other networks succeeded in learning the task. In addition, the simplicity of our datasets did not prevent the transformers from learning when color or texture features were available. The transformers seem to struggle specifically with learning shape.

Comment: How do these authors explain the results of Hermann, Chen & Kornblith (2020) who showed that networks can show a shape-based learning and that texture-bias is due to pre-training?

The results from Herman, Chen & Kornblith (2020) fit well to our results. Among the many factors they analysed, the choice of image augmentations had the strongest effect. Specifically, texture bias is reduced by augmentations that affect local image appearance. These augmentations reduce the alignment between color/texture features and image classes, so our interpretation of the learning dynamics would predict that they should reduce texture bias. The authors also showed that avoiding small random crops increases shape bias. Larger, more central crops mean that it is more likely that the training images will contain a border of the central object – and therefore some shape features. Increasing the occurrence frequency of shape features should make it more likely for the networks to learn these features.

We have included these points in our discussion. The related paragraph reads (lines 594ff.)

> This explanation illustrates why training on stylized images [17], using appearance-changing image augmentation [23], and avoiding very small random crops [23] can help increase the shape bias of deep networks, but still fails to make them sensitive to global shape features [21]. Essentially, gradient descent learning favours features with occurrence statistics that align well with the image class distribution. Image stylization makes textures less predictive of image class, making it more likely that the network learns other features. Appearance-changing augmentations like color distortions and blurring have a similar effect. In contrast, using larger and more central crops means that it is more likely that training images will contain boundary of the central object, increasing the prevalence of shape features. However, the underlying learning dynamics are still the same, so the networks will learn features with favourable occurrence statistics. We hypothesise that local shape features (for example, a specific contour on an object outline) occur more frequently and are more straightforwardly aligned with image classes than global shape features (for example, a specific arrangement of several object parts), biasing the network to local rather than global features.

Comment: Please make the 2x2 grid structure in Fig 1 & 2 (conditions in rows and columns) consistent with from the grid structure in Fig 7, 8, 9 & 10. (Fig 1 & 2 show color in row 1 and texture in row 2, while shape task 1 in column 1 and task 2 in column 2. Fig 7, 8, 9 & 10 use a different scheme).

We thank the reviewer for catching this oversight. We have adjusted the arrangement in figures 7 to 10 to match the one in the first two figures. We thought that it was better to keep the shape tasks split by column, since this is consistent with figures 5 and 6, showing the shape-only results.

Comment: It is not clear to me why Figs 7 & 9 contain comparisons with the 'labels' condition and the authors do not discuss the significance of this trace in any detail. If this is not important, perhaps it is better to just remove this trace.

We have removed the “labels” traces as suggested.

Comment: Experiment 4: I am unable to see how the results in Fig 9 are consistent with the results in Fig 4. For example In Fig 4 (top left), the performance of the network on 'Color only' condition is near

perfect. However, in Fig 9, the NTK similarity with color trained network is lowest. In contrast, in the 'Conflict' condition, the network seems to rely more on shape than color - this seems consistent with Fig 9 (top left). However, if one looks at the 'striped rectangles' condition, the network again performs above chance for shape-only condition. But in Fig 9, the NTK similarity with shape-trained network is lowest. How do the authors explain these results?

Our interpretation of these results rests mainly on the fact that the alignment between the NTKs for the spcConvNet (Fig 9) is very low. Essentially, the features that the network learns do not clearly map onto the color / texture / shape categories in the dataset. Rather, the network learns a mix of features, some of which are related to color/texture, some to shape. This explains why the network performs above chance in all single-feature conditions. However, the exact level of performance on these test sets does not have to match the NTK similarity values, since the similarity only shows how well the learned features align with the shape / color / texture dimensions overall, not how well these features will translate to the test sets.

We have extended our explanation of experiment 4, which hopefully makes this point clearer (lines 429ff.), which now reads

> Thus, the network does not learn color, texture, or shape features for the whole datasets. Instead, it learns a larger mix of features, each of which applies to a subset of images – some of which related to color or texture, others to shape.

Further comments:
l. 78: neither -> none

We have corrected this as suggested.

l. 323-324: "Our results suggest that color and *shape* belong to the former kind of feature, whereas shape belongs to the latter." -> The first shape should be "texture".

We have corrected this line as suggested.

Reviewer 3

Comment: The study focuses on basic geometric shapes, which may not adequately represent the diverse range of shapes encountered in natural environments. The importance of training models on a wide variety of shapes to improve their generalization capabilities is well known. While the authors address that their experiments are simple in the discussion section, it might be compelling to see the dataset include diverse and complex shapes that enhance the robustness of the findings. While I appreciate the use of controlled stimuli for proof of concept, it would add value to confirm that the findings generalize to use of, say, circles or triangles or their combinations and are not limited to rectangle like structures.

We agree that the restricted nature of our datasets is one of the main limitations of our study. This was one of the reasons we used datasets with two different shape types – to show that our results were not limited to one. However, we do see the advantage of using a lager number of different shapes in a single dataset. We have therefore added experiments on a new dataset with a larger range of shapes (rectangles, ellipses, crosses, triangles, and parallelograms). We decided to report these results in an appendix, since the main text would become too long and convoluted otherwise.

Comment: Lines 185-186 : It might be useful to identify some examples of the edge cases that lead to small improvement in shape selectivity. Such examples could even be used to support the findings.

While we agree that it would be interesting to find out how some of the networks manage to be just above chance level, this is difficult to do in practise. The shape-only test sets contain about 10000 images, so if a network has an accuracy of 52% it classifies about 5200 images correctly – but since chance level is 50%, the network could have gotten about 5000 images correctly by random guessing.

We have not found a good way to determine which images the network classifies correctly because of some shape selectivity it has learned, and which ones by chance.

However, we realize that writing about "specific edge cases" without giving any specifics is confusing. We have therefore expanded our explanation slightly and hope that it is now clearer what we mean.

Comment: Lines 316-323: It might be useful to explain it through an intuitive figure or mathematical example, since it helps drive the take-home message clearly.

We have added a simple example and explained in detail how analysis of the learning dynamics would work in this case. We hope that this makes the idea clear.

Comment: While this paper does a good job in using controlled experiments to identify some root causes of why neural networks do not exhibit shape selectivity like humans, I wonder if the paper's findings can shed light on other observable differences/bias between humans and neural networks for related tasks, or are the findings strictly shape selectivity specific?

This is an interesting point. While our results only apply to shape selectivity, our approach may be applicable to other differences between human vision and deep networks as well. We have added this point to our discussion. The corresponding paragraph reads (lines 705ff.)

> While we have only examined the bias for shape, color and texture, there are several other differences between deep networks and human vision [12], such as the susceptibility for adversarial attacks [14] and robustness to image degradation [6]. While our results have to direct bearing on these issues, analyzing the learning dynamics in a similar way is an interesting direction for further work. For example, it has already been shown that networks can learn to be robust to certain image degradations when explicitly trained for them [6], similar to how networks can learn shape-based classification on a shape-only dataset. While designing custom architectures or modified datasets may be sufficient to solve individual problems, understanding how these differences to human vision arise from the learning dynamics of deep networks may point the way to more principled solutions.

Comment: While there is some discussion about how the findings can be related to human visual system, it can be improved further. Explaining and expanding on how "work on combining deep networks with an explicit memory mechanism [49, 50]" in line 515 relates to the architectural and learning algorithm based findings of the paper can help. I assumed the goal of the paper was to bridge the gap between human behavior and that exhibited by neural networks, however, while the paper does a good job of providing some solid reasons of the gap between the two, I could not necessarily connect the findings to a testable solution.

We have extended our explanation of how (we think) memory mechanisms may help enable more shape selective learning. Hopefully, this provides a reasonable next step towards making deep networks more shape selective. Unfortunately, other than this we do not know a direct, simple way to use our results to make networks shape selective, but we hope that our work can help understand the problem better, which is always a good first step towards a solution.
