# Supplementary material for: Teaching deep networks to see shape: Lessons from a simplified visual world
Source: PLoS Comput Biol. 2024 Nov 11;20(11):e1012019. doi: 10.1371/journal.pcbi.1012019 (PMC11581402; doi:10.1371/journal.pcbi.1012019)
Supplement: Attachment — Submitted filename: ResponseToReviewers.pdf [file pcbi.1012019.s003.pdf]

Response to Reviewers

Again, we would like to thank all three reviewers for their kind and insightful comments.

Please find below a point-by-point response to the remaining comments. As before, our responses are highlighted in blue.

Reviewer 1

Comment: The authors have addressed my concerns. I think this is a really nice study.

Thank you.

Reviewer 2

Comment: The authors have done an excellent job in revising the manuscript.

Thank you.

Comment: If they get a chance to revise the manuscript further before publication, my one recommendation will be to more clearly state from the beginning that the networks have to be untrained for them to fail on the shape-only task and pre-trained networks are able to learn this task (e.g. in the Abstract, the authors could say that "we show that some *untrained* networks are unable to learn about shape"; similarly in the Introduction). This is useful information for future research. Other than this, I think the manuscript looks great - it is well written and informative for researchers in the area.

This is a good point. We have added the qualification that the networks have to be trained "from scratch" on the shape only dataset in the abstract and in the introduction (line 94). We chose the formulation "from scratch" since we thought writing about training untrained networks might be confusing to some readers.

Reviewer 3

Comment: The authors did extend their experiment to larger range of shapes. Their results do not suggest big difference between what was previously observed in Experiments 1 and 2. I support the decision to include the results in the supplementary and mentioning it in the discussion section. I think including these additional experiments and comparing the results with results about local-global shape learning capabilities of neural networks from recent studies is interesting but very short. I would have appreciated more discussion on how this connects to natural images, rather than a line mentioned in supplementary.

Thank you for your endorsement. Concerning the brevity, we have kept the discussion of the link to local-global shape processing and natural images short for two reasons. First, we wanted to avoid making the manuscript too long. Second (and more importantly), our results simply do not enable us to draw reliable inferences about these topics. We tried to design the expanded dataset in a way that would enable us to tell apart the contributions of local and global shape features, but did not observe any differences (see lines 462ff.). Any further links we could draw to local-global shape processing in natural images would be purely speculative and open to the type of criticism voiced below (comment 4).

Comment: I understand the difficulty expressed by the authors about identifying "specific edge cases". I appreciate the change of writing and better explaining this part. However, I was expecting that if the same experiment is repeated multiple times and certain images are correctly classified above chance across all the repetitions, then those could serve as potential edge case examples and might have given insights about working of the model, specifically what features lead to more shape selectivity. However, it is quite possible that such images do not exist and the effect is only an aggregate effect. In that case finding singular examples might be hard, I agree.

Yes, unfortunately we were unable to find such a clear pattern. Therefore, we believe the most likely explanation is that some of the color or texture detectors that the networks learn also respond to grayscale edges. This then is enough to slightly bias the responses on the shape-only dataset, as we briefly explained in the manuscript (see line 197ff).

Comment: The example explaining analysis of the learning dynamics is very helpful for any reader who would want to develop an intuition of the learning dynamics. It also benefits readers who may not want to dive deep into the mathematical intricacies of the model.

Thank you. We are happy that we could improve the readability of our manuscript.

Comment: Adding two more observed differences between humans and neural networks is great. However, I hoped there could be examples added with some more experimental simulations involved. Additionally, there are multiple papers in the literature that have explored the mentioned differences of which only one was cited for each. Also, it might not be fair to mention observations from natural image dataset experiments without necessarily testing them for simple shape dataset used in this work. The fact that authors do mention that their results do not have direct bearing to the observations can be considered cautionary, but I would probably remove this addition from the text. It feels half baked, not well referenced (there are already multiple papers on proposing principled solutions) and not relevant for the very simple dataset used here.

We agree that it would be interesting to see experiments that use our methodology to target other differences between human vision and deep networks. However, we think that this is beyond the scope of the manuscript – it would at a minimum require designing yet another dataset with entirely different stimulus dimensions. The main point we wanted to make in the new paragraph (lines 690-702) was that we think such investigations would be promising. We did not mean to make any (half-baked) claims about what our results mean for these problems, since (as we stated) our results do not provide a basis for that. We have revised lines 697-702 to increase the emphasis on this. This part now reads:

> While each of these problems can be solved, for example by designing custom architectures [58, 59] or training on augmented datasets [60, 61], understanding how these differences to human vision arise from the learning dynamics of deep networks may point the way to unified solutions.

We have also added more citations (references 15, 58, 59, 60, and 61).

Comment: Thanks for improving the explanation of how memory mechanisms can enable more shape selectivity.

Thank you.
